# Effectiveness of Organic and Inorganic Fluoridated Dentifrice on Dental Caries Progression Among Institutionalized Geriatrics: A Randomized Intervention

**DOI:** 10.7759/cureus.21058

**Published:** 2022-01-09

**Authors:** Arthi Balasubramaniam, Madan Kumar PD, Kiran Iyer, Dhanraj Ganapathy

**Affiliations:** 1 Community Dentistry, Saveetha Dental College & Hospital, Chennai, IND; 2 Public Health Dentistry, Ragas Dental College and Hospital, Chennai, IND; 3 Public Health Dentistry, Karpaga Vinayaga Institute of Dental Sciences, Kancheepuram, IND; 4 Prosthodontics, Saveetha Dental College & Hospital, Chennai, IND

**Keywords:** cariogram, caries risk, organic fluoride, saliva, institutionalized elderly

## Abstract

Purpose

To assess the effects of two commercially available organic and inorganic fluoridated dentifrices on dental caries progression amongst the institutionalized geriatric population.

Materials and methods

A total of 80 participants were selected and randomly allocated using the coin toss method into two groups, viz. Group I with subjects receiving organic fluoridated dentifrice and Group II with subjects receiving inorganic fluoridated dentifrice. Allocation concealment was done using the Sequentially-Numbered, Opaque, Sealed Envelopes (SNOSE) method. Participants were blinded using analogous dentifrice tubes. They brushed their teeth with the dentifrices twice daily using the modified Bass technique for a period of six months. Their compliance with the intervention was monitored regularly. The outcome measure was susceptibility to dental caries assessed by a cariogram. A single, trained, and calibrated investigator assessed the cariogram at baseline and after six months. Collected data were compiled and analyzed.

Results

The mean age was found to be 67.75 ± 4.1 years, 70.05 ±7.4 years among Group I and Group II participants, respectively. The results showed that the efficacy of avoiding new carious lesions was higher (75.85%) in Group I participants as compared to Group II (73.4%) participants, but no significant difference in the mean cariogram sectors was observed. However, there was a statistically significant reduction in the *Streptococcus (S.) mutans* and *Lactobacillus *colony-forming unit (CFU) (p<0.05) with a considerable increase in salivary pH in Group I participants.

Conclusion

There is a marked increase in the possibility of avoiding new carious lesions with a reduction in *S. mutans* and *Lactobacillus *CFU and an increase in saliva buffer capacity over six months of use of organic fluoride dentifrices. Thus, organic fluoride dentifrice can be an effective agent for institutionalized geriatrics in the prevention of dental caries and oral diseases.

## Introduction

The demographic change with the increase in the elderly population (≥ 60 years) globally has resulted in a marked increase in the number of geriatric institutions. Health becomes a major concern among the elderly in old age homes since it requires special attention on health care delivery [1]. Oral conditions, inclusive of dental caries, periodontal disease, and partial and complete edentulism, are common among geriatrics. These conditions can unfavorably alter dietary consumption and nutritional status thereby affecting general health and quality of life [2]. According to a survey of institutionalized seniors in Delhi, 39.2% remained edentulous, 44.9% had decaying teeth, and 57.9% had extensive periodontal disease [3]. Also, a survey among institutionalized elderly in Chennai showed a 67.3% prevalence of dental caries and a 51.3% prevalence of periodontal disease [4].

This higher frequency of dental caries as well as periodontitis in the elderly suggests that minimizing the oral health strain and enhancing the oral health-related quality of life among institutionalized elders should be a priority. Institutionalized elderly populations have various barriers in availing dental health care services. The barriers include their perception of economic burden to their children in spending on dental treatment, constrained access to dental health services in old age homes [5], fear of dental procedures (injection, noise) [6], transportation difficulties, and lack of knowledge on oral hygiene practices [7]. Therefore, an interest in preventive measures of dental diseases among institutionalized elderly is needed, which may help them overcome these barriers. Evidence has shown a reasonable effect of fluorides in the prevention of dental caries [8]. Fluoridated dentifrices, which are inexpensive and widely available, have been proven to be beneficial in preventing dental cavities [9-10]. Organic fluoride diminishes surface tension, enhances fluoride absorption, and improves remineralization of early caries by its amphiphilic character, which is characterized by hydrophobic and hydrophilic components within a single molecule [11]. Also, organic fluoride reduces dental plaque adhesiveness because of the higher affinity of hydrophilic counter-ions to the enamel surface. Organic fluorides are characterized to spread to all the surfaces in the oral cavity quickly due to their tenside property, showing longer clearance of dental plaque and food particles in the oral cavity. Organic fluorides are found to be highly bacteriostatic and bactericidal since they are strongly glycolytic in nature for three to six hours on its application, thereby reducing acid production in the dental plaque and impeding supragingival plaque growth [12]. The administration of amine fluoride dentifrice and mouth rinse lowered the caries incidence, plaque, and gingival indices in clinical research including people of various ages [13-15]. Though inorganic fluorides have proven to be effective against dental caries with their anti-microbial characteristics, they fail to reduce dental plaque adhesiveness and its clearance due to its very meager affinity of hydrophilic counter-ions to the enamel surface [16].

However, there are no clear trials to show organic fluoride effectively helps in minimizing the risk factors for caries in the elderly population. As a result, the current study was designed to examine the effects of two commercially available organic and inorganic fluoridated toothpaste on dental caries risk factors amongst Chennai's institutionalized elderly community.

## Materials and methods

This experimental study was conducted in an old age institution in East Chennai, Tamil Nadu, India, for a period of about six months, in five stages: sample size estimation, participant recruitment, calibration of the examiner, data collection, and checking for compliance. An interventional randomized study design, in accordance with the Helsinki guidelines on ethical parameters, was adopted and the study was approved by the Institutional Review Board. This study was registered in the Clinical Trials Registry - India (REF/2017/11/015967). The study was clearly explained to the participants and informed consent was obtained.

**Figure 1:**
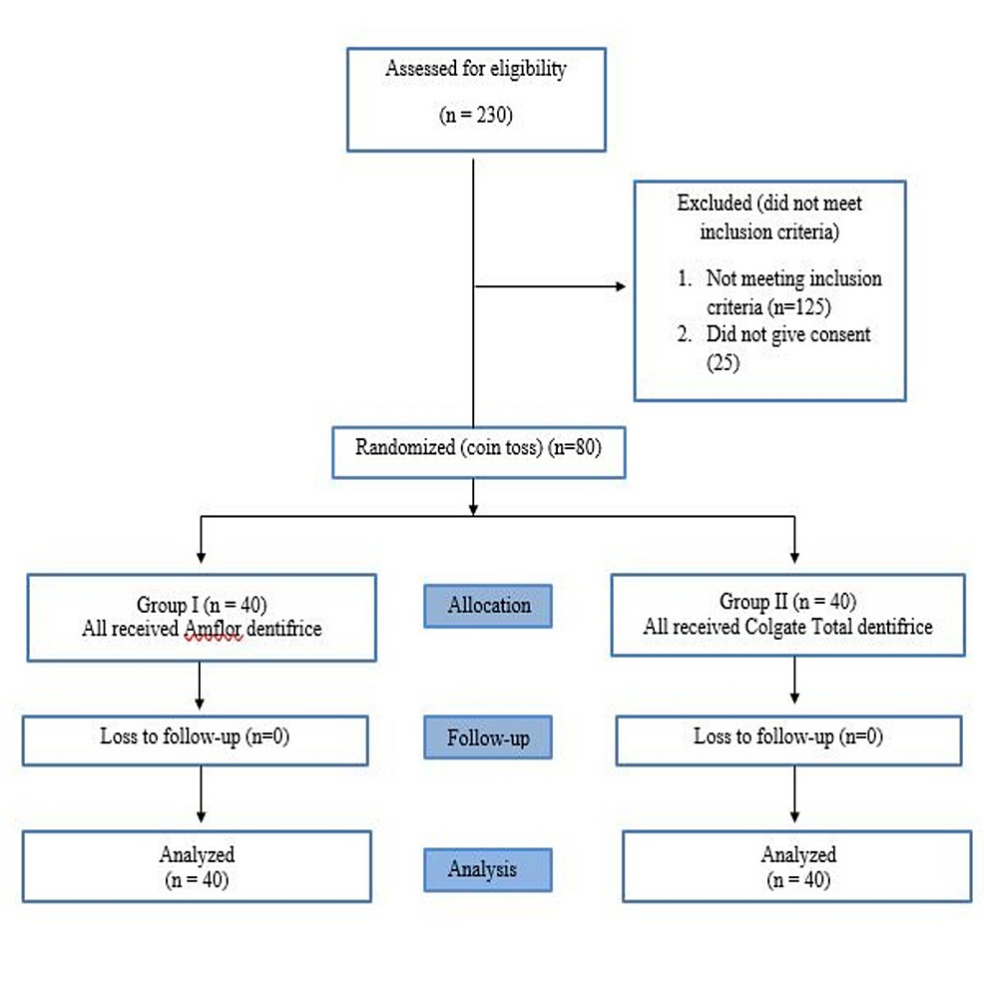
Study Flow Chart

The enrolled respondents were over 60 years old, of both genders, and had lived in an institutional setting for at least a year prior to the beginning of the study. They also intended to stay at the institution for at least another year after the commencement of the research. They possessed a minimum of 10 natural teeth. Participants with uncontrolled diabetes, smoking, severe systemic diseases, and who lacked manual dexterity to perform tooth brushing and availed of any dental services recently were excluded from the study. The sample size was estimated using nPower Software (San Diego, CA) with a margin of error of 5%, which suggested a minimum of 65 participants with 80% power. Sample size was calculated using the formula n = [(Zα/2 + Z β)^2 ^× (S.D)^2^]/ (μ1 - μ2)^2^. To adjust for any potential dropouts, a total of 80 participants were enlisted and randomly allocated using the coin toss method into two groups, viz. Group I (receiving organic fluoridated dentifrice) and 40 participants in Group II (receiving inorganic fluoridated dentifrice). Allocation concealment was done using the Sequentially-Numbered, Opaque, Sealed Envelopes (SNOSE) method. Participants were blinded using analogous dentifrice tubes. They used the dentifrices twice daily once in the morning and night using the modified Bass brushing method as instructed for a period of six months. There was no loss of follow-up (Figure 1). A single investigator, well-trained, and calibrated observed caries and related outcomes and used cariogram software for analysis [17].

**Figure 2:**
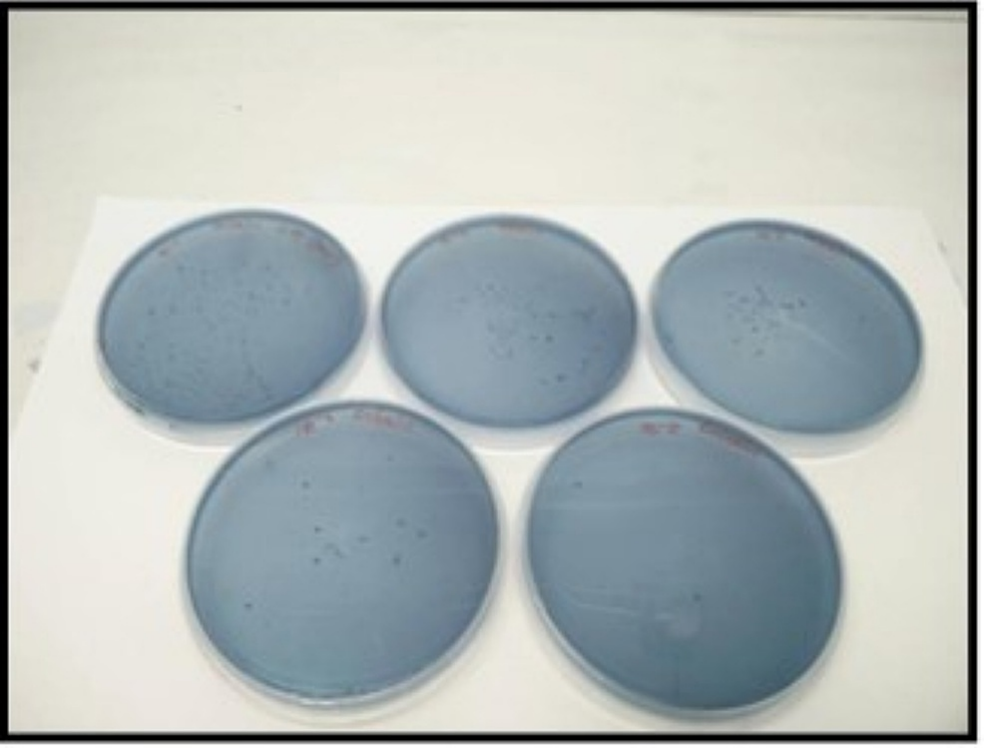
Streptococcus (S.) mutans Culture

**Figure 3:**
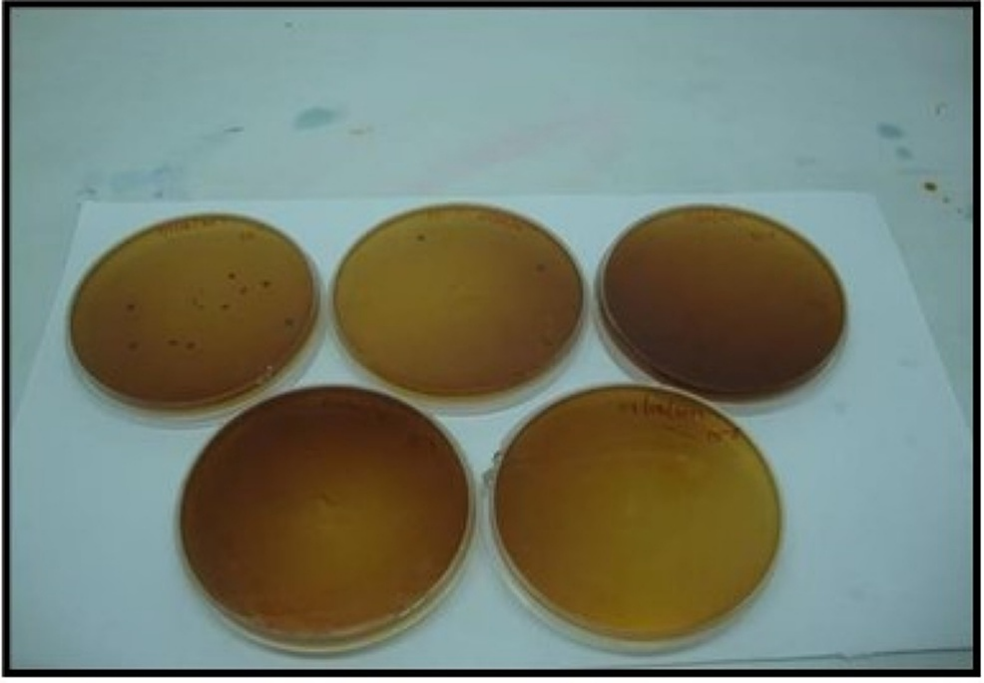
Lactobacillus Culture

The following outcome measures were evaluated. Participants’ demographic profiles and cariogram model factors with a computer-based calculation in the form of a pie chart were investigated. The cariogram model factors include past caries experience, plaque amount, general related diseases, diet content and frequency, *Streptococcus (S.)* ​​​​​​*mutans *count, fluoride program, salivary buffer capacity. The Decayed - Missing - Filled Surface index proposed by Klein, Palmer, and Knutson in 1938 and the plaque index chartered by Silness and Loe in 1964 was recorded for caries experience and plaque accumulation. During a five-minute chewing session on a strip of paraffin wax, stimulated saliva was gathered in the measuring container after two hours of their breakfast. Salivary buffer capacity was assessed using a digital pH meter. The stimulated saliva was cultured in mitis salivarius and Rogosa SL agar for *S. mutans* (MS) (Figure 2) and *Lactobacillus* count (LB) (Figure 3), respectively. The agar substrates were soaked with the stimulated saliva and then incubated for 48 and 96 hours, respectively, at 37 degrees Celsius. The MS and LB counts were divided into four categories, with 0 = 0-10^3^, 1 = 10^3^ -10^4^, 2 = 10^5 ^-10^6^, 3 = > 10^6 ^CFU/ml for MS and 0 = 0-10^2^, 1 = 10^2^ -10^3^, 2 = 10^4^ -10^5^, 3 = > 10^5^ CFU/ml for LB.

To ensure that all of the participants in Groups I and II had the same plaque score, they were given thorough oral prophylaxis before the initiation of the study. After finishing oral prophylaxis, the participants received toothpaste and a toothbrush, and they were instructed to abstain from using other oral hygiene equipment throughout the period of the study. The cariogram model factors listed above were evaluated at baseline and after six months.

Each day, an observation sheet with dawn and night columns was handed to the participants, and they were asked to make a checkmark in the appropriate column after cleaning their teeth. The residential nursing assistant was in charge of this, and the investigator checked up on it once a week. Participants who did not clean their teeth twice a day were strongly advised to brush their teeth twice a day. All the participants enrolled in the study completed it, and there were no dropouts during the study period. All data were analyzed using SPSS, Version 23.0 (IBM Corp., Armonk, NY). A p-value of < 0.05 was considered statistically significant.

## Results

**Figure 4:**
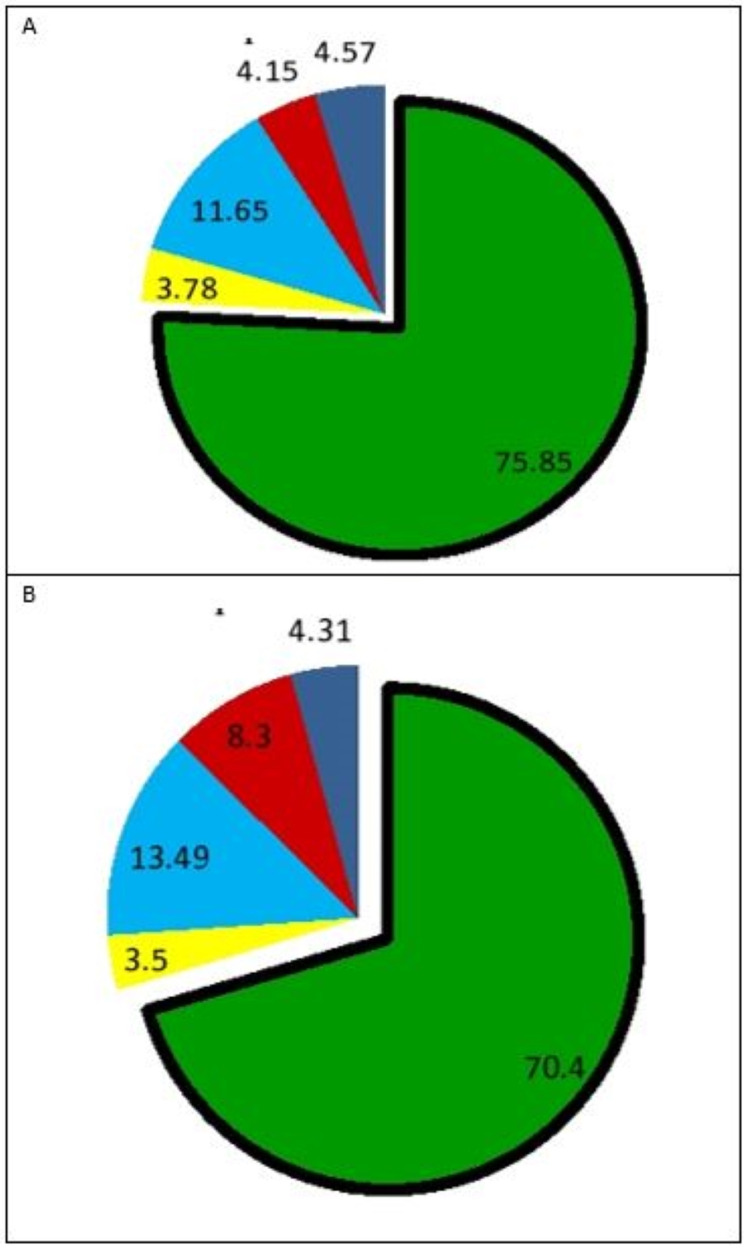
Post-intervention cariogram chart A. For Group I; B. For Group I

The mean age was found to be 67.75 ± 4.1 years; 70.05 ±7.4 years among Group I and Group II participants, respectively. Most of the participants were uneducated in both groups (55%). The participants in both groups had a minimum of 10 natural teeth and a maximum of 32 natural teeth. The comparison of the mean percentage of the green, yellow, red, light blue, and dark blue sectors between the two groups showed no significant difference (p>0.05) (Table 1). However, there was a statistically significant difference in all sectors within the groups (I and II) at baseline and after six months (p <0.05). Figure 4 (A, B) shows the cariogram pie chart at six months for Group I and Group II. The green sector represents the mean chance of avoiding new caries, yellow sector (general diseases), light blue sector (fluoride program and salivary buffer capacity), red sector (*S. mutans* and plaque amount), and dark blue sector (*Lactobacillus* count).

**Table 1:** Comparison of cariogram sectors such as green, yellow, light blue, red, and dark blue between and within two groups * Unpaired t-test; # Paired t-test

Variables	Timeline	Mean ±SD		p-Value
		Group I	Group II	
Green Sector	At baseline	59.93 ± 11.1	59.38 ± 15.7	0.852*
	At 6 months	75.85 ± 8.28	73.40 ±15.6	0.384*
	p-value	0.000^#^	0.000^#^	
Yellow Sector	At baseline	4.15 ± 1.51	3.85 ± 1.49	0.300*
	At 6 months	3.78 ±1.38	3.50 ± 1.41	0.275*
	p-value	0.001	0.011	
Light Blue Sector	At baseline	14.35 ± 9.71	16.93 ± 8.64	0.396*
	At 6 months	11.65 ± 4.11	15.18 ± 7.24	0.137*
	p-value	0.030^#^	0.021^#^	
Red Sector	At baseline	7.55 ± 4.42	11.33 ± 4.82	0.231*
	At 6 months	4.15 ± 4.81	6.03 ± 4.34	0.141*
	p-value	0.000^#^	0.000^#^	
Dark Blue Sector	At baseline	8.83 ± 1.10	15.2 ± 1.23	0.157*
	At 6 months	1.65 ± 1.70	1.40 ± 0.98	0.424*
	p-value	0.000^#^	0.000^#^	

There was a statistically significant high reduction in the mean *S. mutans*, *Lactobacillus* CFU/ml, and plaque score in Group I compared to Group II participants (p <0.05). Also, there was a slight increase in the mean salivary pH in Group I at six months when compared to that of Group II (p <0.05) (Table 2).

**Table 2:** Comparison of mean Streptococcus (S.) mutans, Lactobacillus colony-forming unit (CFU)/ml, plaque score, and salivary pH between and within two groups * Unpaired t-test; # Paired t-test

Variables	Timeline	Mean ±SD		p-Value
		Group I	Group II	
S.mutans	At baseline	9.3x10^5^ ± 3.7x10^5^	5.5x10^5 ^± 2.6x10^5^	0.000*
	At 6 months	1.2x10^3^ ± 0.5x10^3^	3.1x10^3^ ± 1.4x10^3^	0.000*
	p-value	0.000^#^	0.000^#^	
Lactobacillus	At baseline	2.4x10^5^ ± 2x10^5^	3.6x10^5^ ± 2.2x10^5^	0.000*
	At 6 months	7.4x10^2^ ± 1x10^2^	2.5x10^3^ ± 1.4x10^3^	0.000*
	p-value	0.000^#^	0.000^#^	
Plaque score	At baseline	1.69 ± 0.85	1.54 ± 0.97	0.491*
	At 6 months	1.29 ± 0.74	1.21 ±0.78	0.155*
	p-value	0.000	0.000	
Salivary pH	At baseline	6.82 ± 0.31	7.15 ± 0.48	0.001*
	At 6 months	6.93 ± 0.27	6.84 ± 0.38	0.248*
	p-value	0.017	0.000	

## Discussion

The results obtained showed a marked increase in the probability of minimizing the occurrence of new cavities in Group I with organic fluoride compared to Group II using inorganic fluoride dentifrice. This finding was similar to the study that reported the number of new lesions on the upper anterior teeth in the AmF/SnF2 group was 13 compared to 20 new lesions in the NaF group in orthodontic patients [18].

There is a high reduction in salivary cariogenic bacteria such as *S. mutans* and *Lactobacillus* in organic fluoride dentifrice compared to inorganic fluoridated dentifrice. The decrement in CFU/ml observed in the survey was consistent with a trial that observed, over the course of two months, the amine fluoride solution group significantly reduced the mean stimulated salivary CFU/ml of *S. mutans* from 10^5^ to 10^3^ CFU/ml while the sodium fluoride gel group significantly increased the mean stimulated salivary CFU/ml of *Lactobacillus* from 10^5^ to 10^4^ [19].

A six-week clinical investigation involving 34 healthy young adults found that using organic fluoride toothpaste reduced salivary secretion while increasing salivary buffering capacity [20]. Despite the fact that there was no substantial difference in mean saliva secretion rate between the two groups in this study, there was an upsurge in salivary buffering capacity and considerable reduction in the risk of caries as assessed by the cariogram model with the organic fluoride group.

Several studies have shown that amine fluoride is more efficient than sodium fluoride in suppressing tooth plaque and bacterial adhesion [18,21-22]. However, the present study did not observe any marked difference in the mean plaque amount between the two groups. This contradiction could be attributed since plaque accumulation in cariogram software used in this study was assessed using the Silness and Loe method, which relied on optical observation instead of using the disclosing agents.

This study investigated alterations in caries risk in the institutionalized geriatric population after a fluoride-based preventative program. Also, the dispensation and use of the fluoride dentifrices were regularly monitored. This study assessed all the risk factors in the cariogram model. The limitation of this study could be the solitary geriatric institution. Diet, individual susceptibility to tooth degradation due to microtoxins, and salivary modifiers could be the possible confounding variables in the study. However, since the diet was standardized in the geriatric homes and the subjects were selected on strict inclusion criteria and were randomly allocated thereafter, the role of these confounders was deemed negligible. Hence, further extensive multicenter trials in different parts of the globe should be initiated to understand the effects better. Severe metabolic disorders can possibly alter this outcome in the clinical scenario. More extensive interventions can be attempted in the geriatric population with systemic diseases to understand the impact of fluoridated dentifrices in those vulnerable groups.

## Conclusions

The present study throws light on the potential use of organic fluoride for the prevention of the risk factors of dental caries among the institutionalized geriatric population. The overall study results showed that the mean chance to avoid new cavities was high in organic fluoride dentifrice compared to inorganic fluoride dentifrice. There was a reduction in mean cariogenic bacteria *(S. mutans and Lactobacillus)*, the potential risk factor of dental caries, and there was an increase in the salivary buffer capacity on the use of organic fluoride dentifrice compared to the use of inorganic fluoride dentifrice among the geriatric population.
